# 63. PK-RNN-V: A Deep Learning Model for Vancomycin Therapeutic Drug Monitoring using Electronic Health Record Data

**DOI:** 10.1093/ofid/ofab466.063

**Published:** 2021-12-04

**Authors:** Nigo Masayuki, Hong Thoai Nga Tran, Ziqian Xie, Han Feng, Laila Bekhet, Miao Hongyu, Degui Zhi

**Affiliations:** 1 University of Texas in Houston, Houston, TX; 2 School of Biomedical Informatics, University of Texas Health Science Center at Houston, Houston, Texas; 3 School of Public Health, University of Texas Health Science Center at Houston, Houston, Texas; 4 University of Texas Health Science Center, Houston, Texas

## Abstract

**Background:**

Therapeutic drug monitoring (TDM) for vancomycin (VAN) with Bayesian models is recommended by national guidelines. However, limited data incorporating the models may hurt the performance. Our aim is to develop a novel deep learning-based pharmacokinetic model for vancomycin (PK-RNN-V) using electronic medical records (EHRs) data to achieve more accurate and personalized predictions for VAN levels.

**Methods:**

EHR data were retrospectively retrieved from Memorial Hermann Hospital System, comprising 14 hospitals in the greater Houston area. All patients who received VAN and had any VAN levels were eligible. Patients receiving hemodialysis and extracorporeal membrane oxygenation were excluded. Demographic data, vital signs, diagnostic codes, concomitant medications, VAN administration, and laboratory data were preprocessed as longitudinal data. VAN infusion, VAN level measurement, or each hospital day were the time steps for the models. The dataset was splited 70:15:15 for training, validation, and test sets. Our PK-RNN-V model predicted individual patient volume distribution (v) and VAN elimination (k) at each time step using an irregular timesteps GRU model. To compare, Bayesian models were developed from publicly available models, and tuned to feedback the first VAN level to update the v and k. (VTDM)

**Results:**

A total of 12,258 patients with 195,140 encounters were identified from Aug, 2019 and March, 2020. After exclusion of 6,775 patients, 5,483 patients with 8,689 encounters were included. Table 1 summarized the characteristics of patients included in our study. 55,336 doses of VAN were administered with a median dosage of 1.0 gm. VAN levels were measured 18,588 times at various timings. The median VAN level was 14.7 mcg/mL Table 2 described the performance of our models and VTDM models. Our model exhibited better performance compared to VTDM model (RMSE: 5.64 vs. 6.57, respectively). Figure 1 shows example prediction curves of VAN levels from each model.

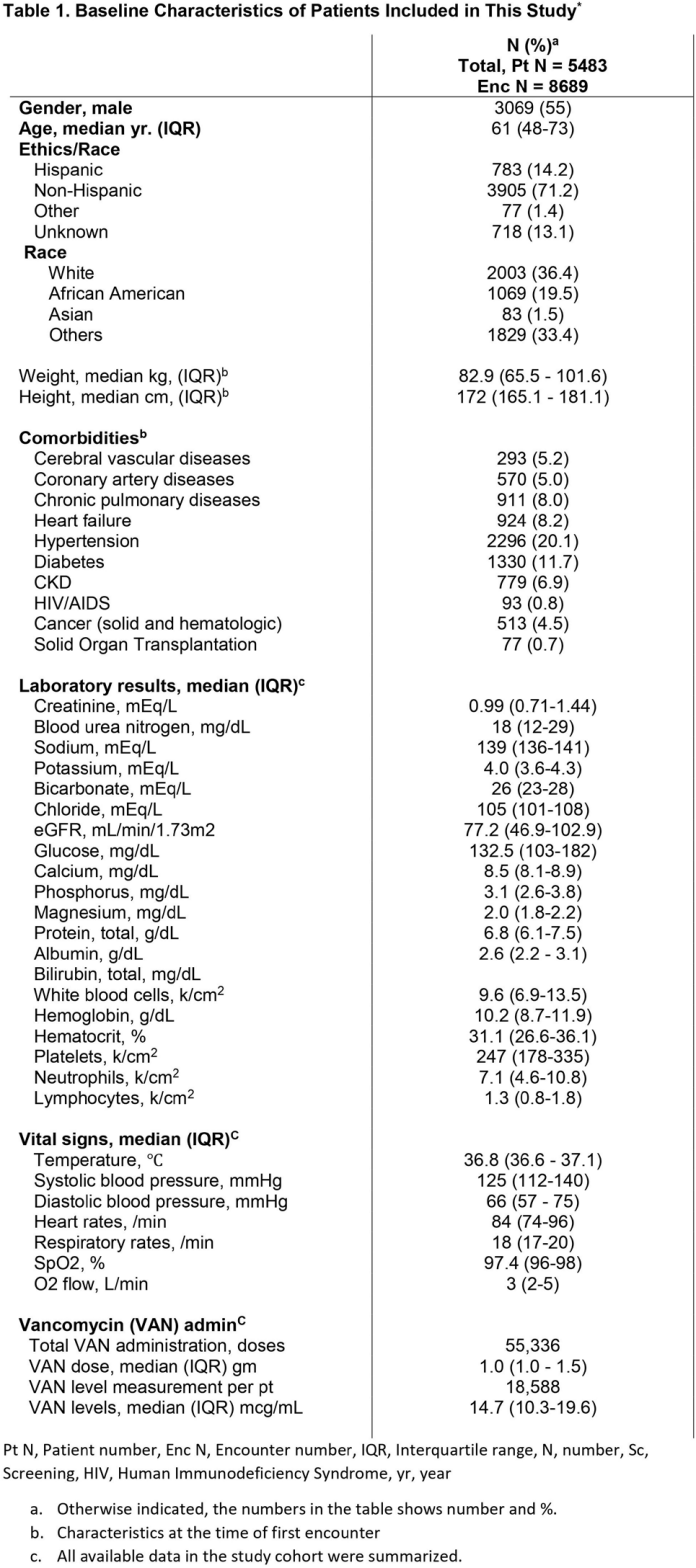

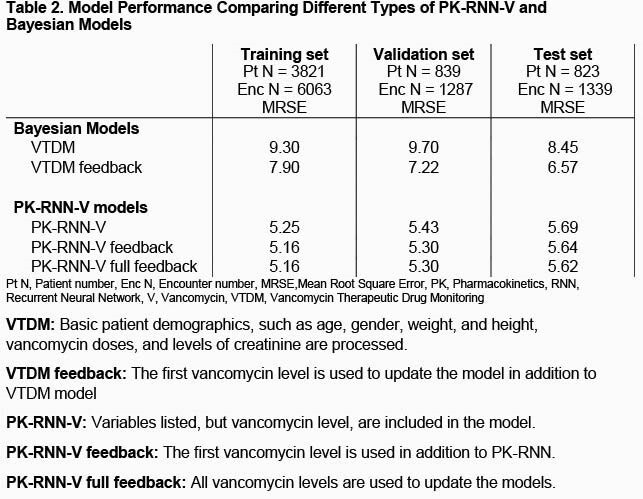

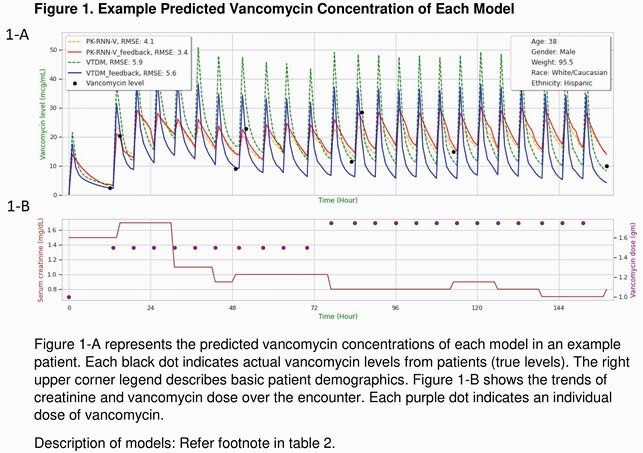

**Conclusion:**

PK-RNN-V model is a novel approach to predict patient PK and VAN levels. Our results revealed promising performance of this model. Our model can take a wide range of real-world patient data into the model. Further studies are warranted for external validations and model optimizations.

**Disclosures:**

**All Authors**: No reported disclosures

